# Determining target populations for leprosy prophylactic interventions: a hotspot analysis in Indonesia

**DOI:** 10.1186/s12879-022-07103-0

**Published:** 2022-02-07

**Authors:** A. T. Taal, D. J. Blok, A. Handito, S. Wibowo, Sumarsono, A. Wardana, G. Pontororing, D. F. Sari, W. H. van Brakel, J. H. Richardus, C. R. S. Prakoeswa

**Affiliations:** 1 NLR, Amsterdam, The Netherlands; 2 Department of Public Health, Erasmus MC, University Medical Center Rotterdam, Rotterdam, The Netherlands; 3 Department of Infectious Disease, Leprosy Control Programme, Ministry of Health, Jakarta, Indonesia; 4 East Java Provincial Health Office, Surabaya, Indonesia; 5 NLR Indonesia, Jakarta, Indonesia; 6 Department of Dermatology and Venereology, Faculty of Medicine, Universitas Airlangga, Dr. Soetomo General Academic Hospital, Surabaya, Indonesia

**Keywords:** Leprosy, Post-exposure prophylaxis, Geospatial, Hotspots, Targeted interventions

## Abstract

**Background:**

Leprosy incidence remained at around 200,000 new cases globally for the last decade. Current strategies to reduce the number of new patients include early detection and providing post-exposure prophylaxis (PEP) to at-risk populations. Because leprosy is distributed unevenly, it is crucial to identify high-risk clusters of leprosy cases for targeting interventions. Geographic Information Systems (GIS) methodology can be used to optimize leprosy control activities by identifying clustering of leprosy cases and determining optimal target populations for PEP.

**Methods:**

The geolocations of leprosy cases registered from 2014 to 2018 in Pasuruan and Pamekasan (Indonesia) were collected and tested for spatial autocorrelation with the Moran’s I statistic. We did a hotspot analysis using the Heatmap tool of QGIS to identify clusters of leprosy cases in both areas. Fifteen cluster settings were compared, varying the heatmap radius (i.e., 500 m, 1000 m, 1500 m, 2000 m, or 2500 m) and the density of clustering (low, moderate, and high). For each cluster setting, we calculated the number of cases in clusters, the size of the cluster (km^2^), and the total population targeted for PEP under various strategies.

**Results:**

The distribution of cases was more focused in Pasuruan (Moran’s I = 0.44) than in Pamekasan (0.27). The proportion of total cases within identified clusters increased with heatmap radius and ranged from 3% to almost 100% in both areas. The proportion of the population in clusters targeted for PEP decreased with heatmap radius from > 100% to 5% in high and from 88 to 3% in moderate and low density clusters. We have developed an example of a practical guideline to determine optimal cluster settings based on a given PEP strategy, distribution of cases, resources available, and proportion of population targeted for PEP.

**Conclusion:**

Policy and operational decisions related to leprosy control programs can be guided by a hotspot analysis which aid in identifying high-risk clusters and estimating the number of people targeted for prophylactic interventions.

**Supplementary Information:**

The online version contains supplementary material available at 10.1186/s12879-022-07103-0.

## Background

The chronic infectious disease leprosy is caused by the slowly multiplying *Mycobacterium leprae* that affects the skin and peripheral nerves. The bacteria are mainly transmitted human-to-human through aerosols by coughing and sneezing. It can take on average 2 to 5 years before the first clinical signs or symptoms appear. As long as the infection is undiagnosed and untreated, individuals who carry sufficient bacteria can spread the disease to others. Moreover, delay in diagnosis and lack of treatment can lead to lifelong disabilities and social discrimination. Therefore, besides early diagnosis and treatment of the patient to prevent disabilities, prophylactic treatment to persons at risk of leprosy prevents further transmission of the bacteria [1, 2].

Worldwide, 202,185 new cases of leprosy were detected in 2019 of which around 80% were found in India, Brazil, and Indonesia. Although some decrease is seen in the number of new cases in the last five years, there is strong indication of ongoing transmission as reflected by the 15,000 new child cases [3]. Current elimination strategies are focused on reducing the number of new cases substantially by early detection and providing post-exposure prophylaxis (PEP) to at-risk populations [4]. These at-risk populations are identified as the close contacts of leprosy patients and people that live in the same area. The LPEP study demonstrated the feasibility of PEP interventions among close contacts in seven countries [5]. Moreover, modelling studies predict a long-term impact of PEP interventions on the leprosy trend. The higher the number of contacts per leprosy case provided with PEP, the larger the reduction in incidence trend is predicted [6]. Even a larger impact on the leprosy trend is expected if 1 to 3 rounds of mass chemoprophylaxis are implemented in the total population in addition to close contact strategies [7]. Scaling up PEP interventions and targeting at-risk populations in high-transmission areas would help to further reduce leprosy incidence.

It is difficult to measure the transmission of *M. leprae* directly. Therefore, it is assumed that areas where leprosy cases are located close to each other represent foci of transmission. These (high) transmission areas can be identified by clustering of patients through Geographic Information Systems (GIS). The utilization of GIS is limited in leprosy control programs but is perceived as important for leprosy control and elimination [8, 9]. Understanding the distribution of disease and its relation to environmental, climatological, socioeconomic and health system-related factors has been the primary focus of research. On the other hand, those involved in public health policy and management would be more interested in utilization of GIS for operational purposes, including identifying which areas and population should be targeted for (enhanced) leprosy control and preventive interventions and where the leprosy health services need strengthening [10–13].

The Global Moran’s I statistic and Local Moran statistic (Local Index of Spatial Association [LISA]) have been used at global and local level to highlight areas that are at the highest risk of leprosy occurrence [14, 15], and low socioeconomic level [16]. These statistics calculate the strength of spatial autocorrelation and dependence of indicators such as the annual new detection rate (NCDR), the detection rate of children below 15 years, the detection rate of disability grade 2 (DG2) and socioeconomic indicators. Kernel density estimation is also often used to identify areas with greater intensity of leprosy cases, called hot spots [17–19]. This method calculates the density of points in an area around those points wherein the population in that area can be considered. The resulting maps show hot spots, areas with a high density of cases. Kulldorf’s spatial scan statistic identifies the most likely cluster(s) of cases [20]. The SaTScan [21] has the added value to scan for space–time clustering and is often used in studies to monitor leprosy transmission and clustering over a period [22–25].

Only a few spatial studies have been used to help inform the design and target population for leprosy interventions [9, 13, 26, 27]. For example, the Anti-Leprosy Campaign of Sri Lanka used real-time GIS-based disease surveillance to target and plan leprosy screening programs at street level [13]. In the municipality of Mossoró in Brazil, GIS was used to identify areas with a high leprosy burden to target active case finding campaigns. The four two-week targeted campaigns led to the diagnosis of 104 new leprosy cases which is 90% of the total number of new cases detected throughout the previous year [26]. Barreto et al. (2015) focused on the most likely leprosy cluster and targeted a surveillance intervention by performing PGL-1 serology to determine subclinical infection among healthy contacts of leprosy patients and schoolchildren resident in that cluster [27].

Similarly, spatial data could also be used to identify target populations for prophylactic interventions. Such an application has not yet been established in leprosy control. In other infectious diseases, however, GIS technology is used to improve disease prevention efforts in several ways, for example, by planning the interventions to maximize reach, effectiveness, and efficiency, and by selecting the most promising settings for the interventions [28–30]. In view of the increasing utilization of spatial analysis to identify clustering of leprosy cases, this study aims to establish a GIS-based methodology for leprosy programs to optimize the effectiveness and efficiency of their control activities by identifying clustering of leprosy cases and to determine optimal target populations for prophylactic interventions.

## Methods

### Study area

The present study used spatial data of registered leprosy patients in Pamekasan, Pasuruan regency and Pasuruan city, East-Java province, Indonesia. Although East Java has reached nationwide elimination of leprosy with 73 new cases per 1,000,000 population in 2019, there are regencies where leprosy is still endemic [31]. Pasuruan regency and Pasuruan city are located in East Java, southeast of Surabaya, and together have an area of 1509 km^2^ and a population of almost 1.83 million in 2019 [32]. We considered Pasuruan regency and Pasuruan city as one area for the purpose of the hotspot analysis because, epidemiologically, they constitute one contiguous area (hereafter referred to as ‘Pasuruan’). Looking across administrative areas for purposes like hotspot analyses will help local governments improve their ongoing collaboration and help them in making joint operational decisions related to the leprosy control program in the future. Pamekasan regency is located on Madura Island and has an area of 792 km^2^ and a population of around 880,000 in 2019 [32]. Both Pamekasan and Pasuruan are considered high endemic for leprosy with a new case detection rate (NCDR) of 107 per 1,000,000 population in Pasuruan and of 268 in Pamekasan [31].

### Data collection

Patient information on medical records of all leprosy patients registered from January 2014 to December 2018 was requested from all Primary Health Care Centers (PHCs) in Pasuruan and Pamekasan. The patient information included name, address, gender, age, date of diagnosis, and type of leprosy. Local research assistants travelled together with health volunteers to the residence of the patient and to collect the coordinates of residence (longitude and latitude) using the mobile application MapIt (version 7.6.0, https://mapitgis.com/), a tool for Geographic Positioning Systems (GPS) data collection and management. The GPS coordinates were uploaded daily to a server in Jakarta. Serial numbers were randomly appointed to each data point to anonymize the data. All the anonymized data points were processed and verified using the open-source Quantum Geographic Information System (QGIS) version 3.4.1 (QGIS Developer team, Madeira (2018)). Incorrectly georeferenced points (e.g., located in water bodies, open-field) were recollected. Population data of Pasuruan from 2017 [33] and of Pamekasan from 2015 [34] were obtained from Baden Pusat, Republik Indonesia at https://pasuruankab.bps.go.id and https://pamekasankab.bps.go.id, respectively.

### Spatial analysis

Detailed maps were constructed with the spatial data to visualize the leprosy distribution for Pamekasan and Pasuruan using QGIS. The spatial data was combined with the patient information as retrieved from the medical records, and population data. The number of cases per subdistrict and the new case detection rate (NCDR) per 1,000,000 population was calculated for each area.

The presence and strength of clustering of new leprosy cases was calculated using Univariate Moran’s Index statistic [35] in ClusterSeer program version 2.5.2 (BioMedware.Inc, https://biomedware.com). The Moran’s Index measures the correlation coefficient between spatial points, where zero indicates homogenous distribution (no clustering) and a value close to + 1 or – 1 indicates clustering or dispersed distribution, respectively.

The Heatmap tool of QGIS was applied to identify clusters of new leprosy cases. Heatmaps provide an estimation of the density of data in an area of interest. The tool draws a circle around each data point (i.e., leprosy case) and uses Kernel Density Estimation to create a density (heatmap) raster of all data points. For each raster cell, it calculates the density of points by measuring the distance to one or more data points. A raster cell directly on top of the data point has a value of 1 and this value decreases with the distance to the point, whereas a cell outside the circle (i.e., area not of interest) has a value of 0. For raster cells that fall into multiple circles, the tool sums up all distances to each point. This result in higher values. A radius needs to be chosen that will specify the circle around each point. A large radius will result in greater smoothing because of more overlapping circles and thus more raster cells with high vales, whereas a smaller radius will result in finer details of clusters. The result will be a raster map that shows pockets of ‘heat’, where there is a high concentration of points (leprosy cases).

To find the most effective use of the heatmap to identify clusters to target a PEP intervention, we selected five different radii: 500 m, 1000 m, 1500 m, 2000 m and 2500 m, and the Kernel shape quartic weight with raw values as a density measure. The strength of clustering is determined by the density of points and the higher the value the stronger the clustering. In this study, we selected three cut-off values for the density of points to define a cluster as low (≥ 2), moderate (≥ 5), and high density (≥ 10). In general, fewer clusters of cases were identified when we only applied the cut-off for high-density clustering. We used the three cut-off values in combination with the five different radii, resulting in 15 cluster settings. For each cluster setting, we calculated the number of cases per cluster, the cluster area in km^2^ and the total population in clusters. The total population in clusters was calculated by multiplying the total cluster area by the population density.

Next, we calculated the total target population for PEP intervention for three intervention strategies: (I) providing PEP to 20 contacts per index case in a cluster (i.e., standard contact strategy as recommended by WHO [4]); (II) providing PEP to 100 individuals per index case in a cluster; and (III) providing PEP to the total population in a cluster (i.e., population-wide or blanket strategy). For each cluster setting, the total population targeted for PEP was calculated by multiplying the total number of cases in a cluster by 20 for strategy I and by 100 for strategy II. Then, this number was divided by the total population in clusters to give the proportion of population targeted for PEP. In some cluster settings, this proportion can exceed 100% because the total population in a cluster may be smaller than the calculated target population.

### Sensitivity analysis

The accuracy of our approach to identify clusters was assessed through a sensitivity analysis. We repeated our spatial analysis using data of leprosy cases registered from 2014 until 2016 (i.e., three years of data) only. We identified clusters of leprosy cases for all 15 cluster settings. Afterwards, we calculated the proportion of cases registered in 2017 and in 2018 that would fall in the identified clusters for each cluster setting by dividing the number of cases in clusters by the total number of cases in year 2017 or 2018.

### Ethics

The research protocol was approved by the ethical review board at the National Institute of Health Research and Development of the Ministry of Health in Indonesia (#LB.02.01/2/KE.400/2019) and the Ethical Committee in Health Research at Dr. Soetomo General Hospital Surabaya in Indonesia (#1369/KEPK/VIII/2018). For this study, patient’ information has been retrieved from the national register and a copy of the dataset has been anonymized to perform the hotspot analysis by the first author. The consent procedures will be followed the moment patients will be approached to participate in the PEP++ project.

## Results

### Distribution of cases

**Fig. 1:**
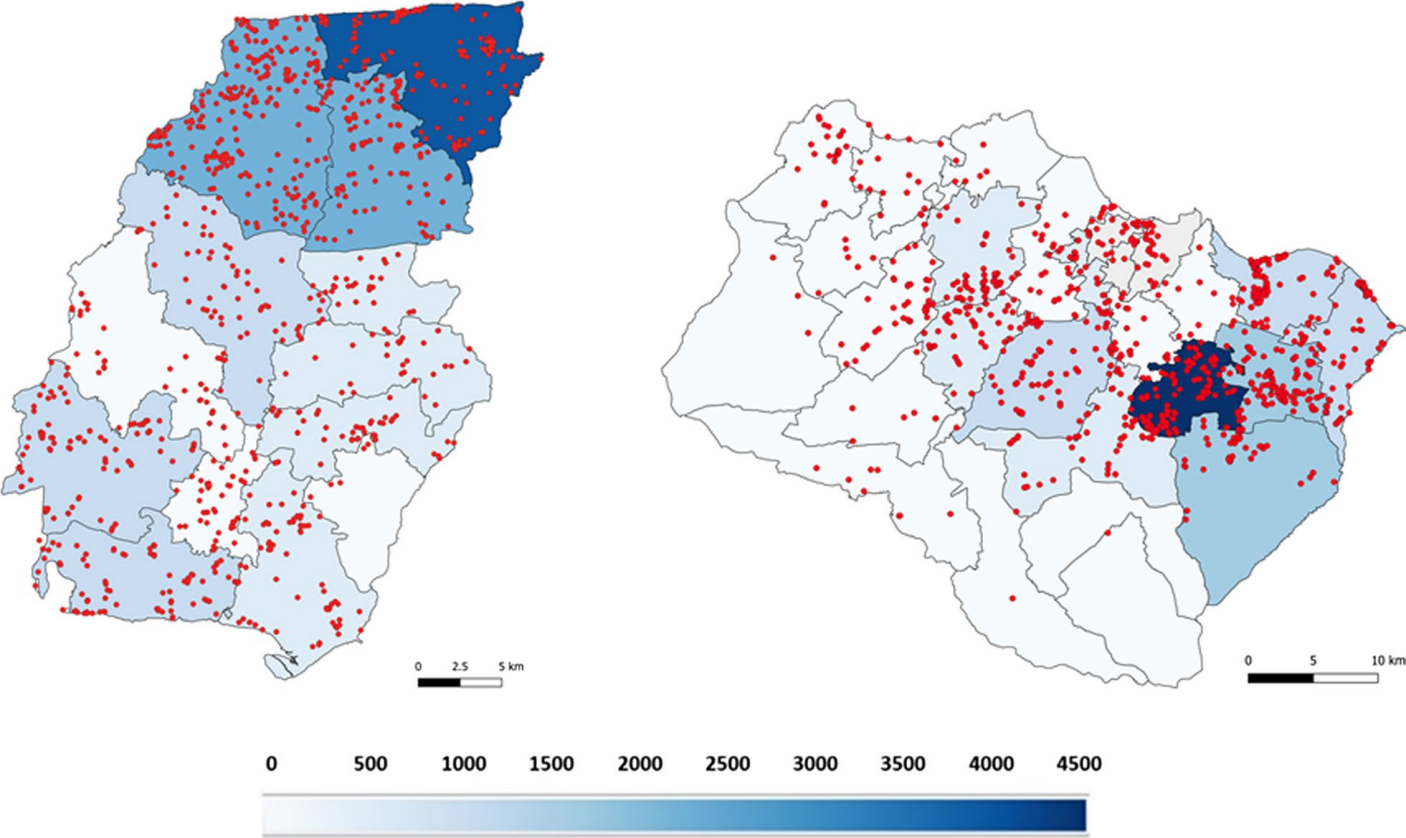
Spatial distribution of leprosy in Pamekasan and Pasuruan district. Spatial distribution of leprosy cases in Pamekasan (left) and Pasuruan district (right). The maps present the new case detection rate per 1,000,000 population for each subdistrict (blue scale). The red points are the location of mapped leprosy patients

From 2014 to 2018, 1080 new leprosy patients were registered at the PHCs in Pasuruan and 1244 in Pamekasan district. The locations of 1056 (98%) and 1142 (92%) patients’ houses were georeferenced in Pasuruan and Pamekasan, respectively. Figure 1 shows the distribution of the leprosy cases in both areas and the NCDR of each subdistrict as calculated as the number of cases from 2014 to 2018 per 1,000,000 population. The NCDR ranged from 173 to 3901 per 1,000,000 population in Pamekasan and from 19 to 4496 per 1,000,000 population in Pasuruan. The northern subdistricts in Pamekasan show a higher NCDR compared to the southern subdistricts. In Pasuruan, subdistricts with the highest NCDR are found in the eastern part of the district. The Moran’s I value calculated for Pamekasan was 0.27 with a p-value of 0.002 and a z-score of 6.34. For Pasuruan, this was 0.44 with a p-value of 0.002 and a z-score of 15.04. The positive Moran’s I value and z-score both indicating clustering of leprosy cases.

### Cluster maps of the 15 different cluster settings

**Fig. 2:**
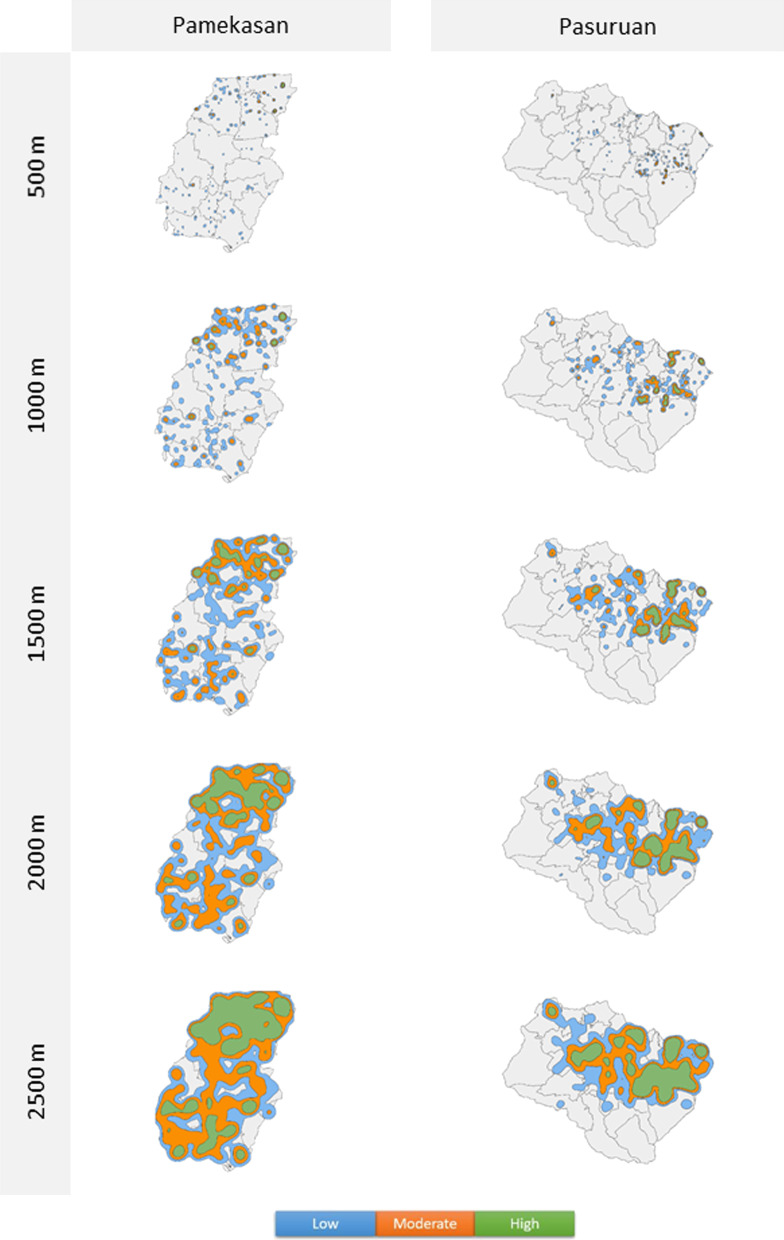
Clusters in Pamekasan and Pasuruan by heatmap radius and cluster density. Cluster maps of Pamekasan (left) and Pasuruan (right) by heatmap radius and cluster density. Heatmap radius varies from 500 m (top row) to 2500 m (bottom row). The blue colour represents low density clusters (≥ 2), the orange colour represents moderate density clusters (≥ 5), and the green colour represents high density clusters (≥ 10)

Figure 2 shows the clusters with the different heatmap radius and cluster density for Pamekasan and Pasuruan. The clusters were evenly distributed through Pamekasan with more high density clusters in the north. In Pasuruan, the clusters were focused in the central and north-eastern part of the area. Using a radius of 500 m, 1000 m and 1500 m resulted in many individual clusters while 2000 m and 2500 m in a few large, smoothed clusters. Clusters based on a 500 m and 1000 m radius had more low density clusters than high density clusters, whereas clusters based on a 2500 m radius resulted in more high density clusters.

### Proportion of cases in clusters and population targeted for PEP

**Fig. 3:**
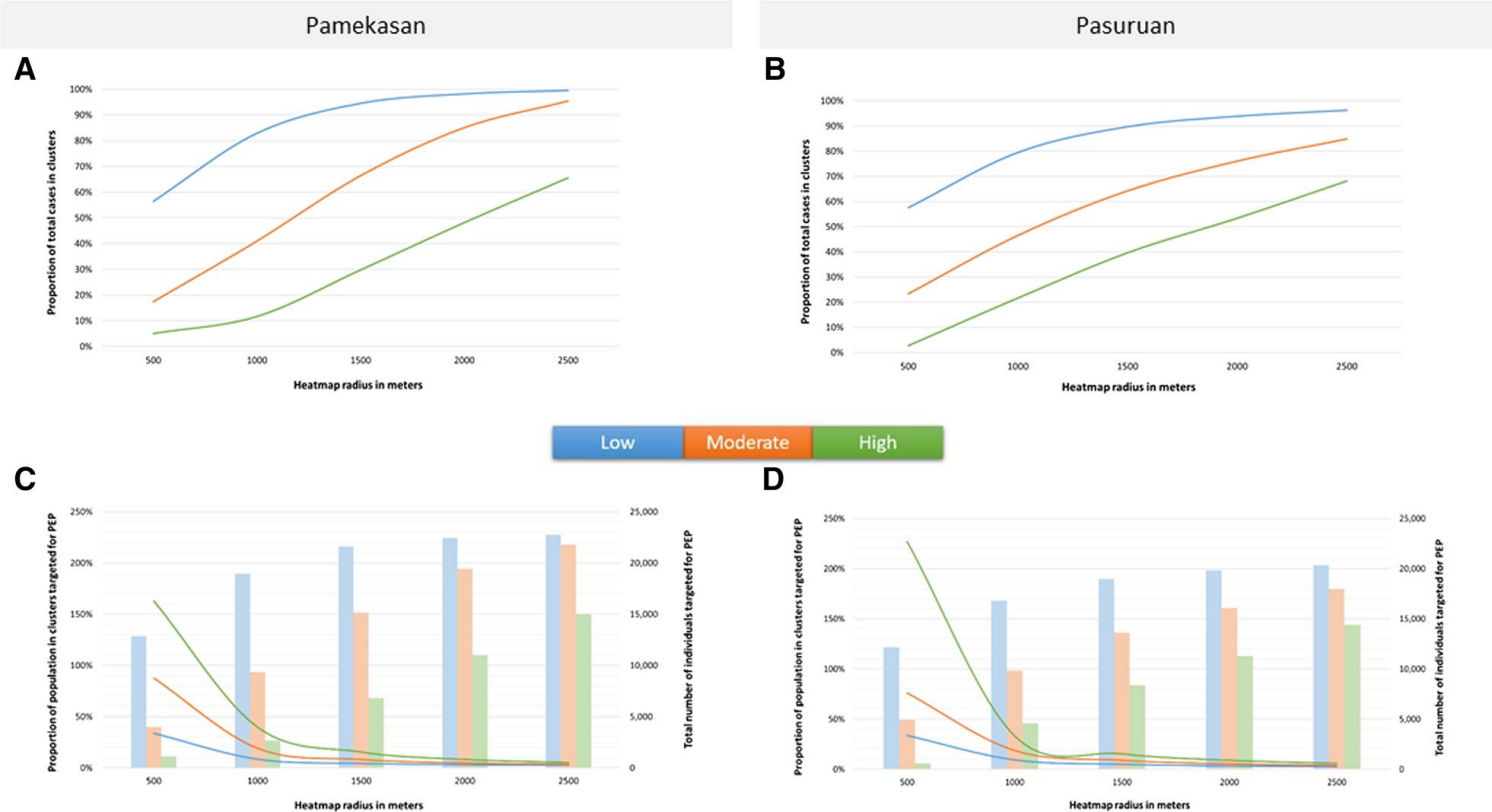
Proportion of cases in clusters and population targeted for PEP by radius and cluster density. The proportion of cases in clusters, the proportion of population in clusters targeted for PEP and the total number of individuals targeted for PEP by radius and cluster density in Pamekasan and Pasuruan. **A** and **B** show the proportion of total cases in clusters in Pamekasan and Pasuruan. **C** and **D** show the proportion of population in clusters targeted for PEP (line) and the total number of individuals targeted for PEP if 20 contacts per leprosy case will be targeted (bars) by radius and cluster density in Pamekasan and in Pasuruan, respectively. The blue colour represents low density clusters, orange moderate density clusters, and green high density clusters

In Pamekasan, the proportion of cases in clusters increased by heatmap radius from 56.4 to 99.6% for low density clusters, from 17.4 to 95.4% for moderate density clusters, and from 5.0 to 65.6% for high density clusters (Table 1 and Fig. 3A). Also, in Pasuruan, the proportion of cases in clusters increased by heatmap radius from 57.5 to 96.2% for low density clusters, from 23.4 to 84.9% for moderate density clusters, and from 2.7 to 68.1% for high density clusters (Table 1; Fig. 3B). The proportion of population targeted for PEP if the intervention strategy of 20 contacts per leprosy case is selected, decreased by heatmap radius, and ranged from 163% (500 m radius and high density clusters) to 3% (2500 m radius and low density and moderate density clusters) in Pamekasan (Table 1; Fig. 3C), and ranged from 227% (500 m radius and high density clusters) to 3% (2500 m radius and low density clusters) in Pasuruan (Table 1; Fig. 3D). If the intervention strategy of 100 contacts per leprosy case is selected, the proportion of population targeted for PEP is five times higher and ranged from 814 to 13% in Pamekasan, and from 1135 to 13% in Pasuruan (Table 1). On the other hand, by increasing the radius, the absolute number of individuals that would be targeted for PEP increases in both districts, with the steepest increase in the high density clusters (Fig. 3C, D).

**Table 1:** The total population targeted for PEP for the three PEP strategies for Pamekasan and Pasuruan

	Heatmap radius (m)	Cluster density^1^	Number of clusters	Total cluster area (km^2^)	Total number of cases in cluster	Proportion of cases in cluster^2^	Population targeted for PEP				
							Contact strategy				Population-wide strategy
							20 contacts		100 individuals		Total population in clusters^3^
Pamekasan	500	Low	122	34.46	644	56.4%	12,880	34%^4^	64,400	168%^5^	38,289
		Moderate	29	4.09	199	17.4%	3980	88%	19,990	438%^5^	4544
		High	3	0.63	57	5.0%	1140	163%^5^	5700	814%^5^	700
	1000	Low	63	201.47	947	82.9%	18,940	8%	94,700	42%	223,853
		Moderate	40	44.05	468	41.0%	9360	19%	46,800	96%	48,944
		High	8	6.01	133	11.6%	2660	40%	13,300	199%^5^	6678
	1500	Low	14	447.46	1080	94.6%	21,600	4%	108,000	22%	497,173
		Moderate	32	168.33	758	66.4%	15,160	8%	75,800	41%	187,031
		High	19	39.33	340	29.8%	6800	16%	3400	78%	43,700
	2000	Low	4	653.95	1122	98.2%	22,440	3%	112,200	15%	726,604
		Moderate	17	366.67	971	85.0%	19,420	5%	97,100	24%	407,407
		High	13	119.3	550	48.2%	11,000	8%	55,000	41%	132,554
	2500	Low	1	789.68	1137	99.6%	22,740	3%	113,700	13%	877,413
		Moderate	4	574.33	1089	95.4%	21,780	3%	108,900	17%	638,138
		High	11	254.6	749	65.6%	14,980	5%	74,900	26%	282,886
Pasuruan	500	Low	86	32.39	608	57.5%	12,160	34%	60,800	169%^5^	35,989
		Moderate	27	5.84	247	23.4%	4940	76%	24,700	381%^5^	6489
		High	3	0.23	29	2.7%	580	227%^5^	2900	1135%^5^	256
	1000	Low	52	161.46	840	79.5%	16,800	9%	84,000	47%	179,398
		Moderate	24	47.13	492	46.5%	9840	19%	49,200	94%	52,366
		High	10	12.29	229	21.7%	4580	34%	22,900	168%^5^	13,655
	1500	Low	24	347.05	948	89.7%	18,960	5%	94,800	25%	385,607
		Moderate	19	136.44	679	64.2%	13,580	9%	67,900	45%	151,598
		High	10	48.62	419	39.6%	8380	16%	41,900	78%	54,022
	2000	Low	10	541.19	992	93.9%	19,840	3%	99,200	16%	601,316
		Moderate	9	268.94	804	76.1%	16,080	5%	80,400	27%	298,819
		High	11	112.25	564	53.4%	11,280	9%	56,400	45%	124,721
	2500	Low	3	697.94	1017	96.2%	20,340	3%	101,700	13%	775,481
		Moderate	4	418.42	897	84.9%	17,940	4%	89,700	19%	464,906
		High	10	215.44	720	68.1%	14,400	6%	72,000	30%	239,375

^1^Cluster density based on the cut-off values ≥ 2 (low density), ≥ 5 (moderate density), and ≥ 10 (high density)

^2^The proportion of total leprosy cases that living in the area and within a cluster

^3^The total population in clusters was calculated as the cluster area multiplied by the population density of Pamekasan (i.e., 1111.1) and of Pasuruan (i.e., 1212.7)

^4^The proportion of population living in clusters that would be targeted for PEP

^5^The proportion of population targeted for PEP (i.e., number of cases in clusters multiplied with 20 contacts or 100 individuals) exceeds the size of the population in the identified clusters

Table 2 shows the findings of our sensitivity of the spatial analysis. The proportion of cases in 2017 that belongs to identified clusters (based on 2014–2016) increased with heatmap radius and ranged from 3% (500 m and high density clusters) to 93.9% (2500 m; low) in Pamekasan, and 0% (500 m; high) to 81.9% (2500 m; low) in Pasuruan. In 2018, the proportion of cases in the identified clusters also increased with heatmap radius and ranged from 0% (500 m; high) to 95.9% (2500 m; low) in Pamekasan, and 0% (500 m; high) to 74.1% (2500 m; low) in Pasuruan. Additional file 1:Fig. S2 shows the identified clusters of cases based on registered from 2014 to 2016 in Pamekasan and Pasuruan.

**Table 2:** Sensitivity analysis

	Heatmap radius (m)	Cluster density^1^	Number of clusters	Number of cases in cluster	Number cases in clusters		Proportion of new cases in the 2014–2016 clusters for:	
			2014–2016	2014–2016	2017	2018	2017 (%)	2018 (%)
Pamekasan	500	Low	77	354	52	25	19.8	20.7
		Moderate	11	96	16	1	6.1	0.8
		High	2	29	8	0	3.0	0.0
	1000	Low	70	566	122	53	46.4	43.8
		Moderate	24	208	30	16	11.4	13.2
		High	5	62	15	1	5.7	0.8
	1500	Low	26	677	191	79	72.6	65.3
		Moderate	24	208	80	44	30.4	36.4
		High	9	120	22	7	8.4	5.8
	2000	Low	8	727	225	108	85.6	89.3
		Moderate	21	519	152	68	57.8	56.2
		High	9	207	42	27	16.0	22.3
	2500	Low	1	751	247	116	93.9	95.9
		Moderate	10	632	194	87	73.8	71.9
		High	14	312	85	49	32.3	40.5
Pasuruan	500	Low	52	302	48	22	21.2	10.9
		Moderate	13	94	8	4	3.5	2.0
		High	0	0	0	0	0.0	0.0
	1000	Low	36	444	101	71	44.7	35.3
		Moderate	18	225	48	27	21.2	13.4
		High	7	75	9	5	4.0	2.5
	1500	Low	14	527	146	107	64.6	53.2
		Moderate	12	333	92	63	40.7	31.3
		High	9	172	32	24	14.2	11.9
	2000	Low	8	564	167	124	73.9	61.7
		Moderate	11	426	121	90	53.5	44.8
		High	9	268	77	53	34.1	26.4
	2500	Low	5	584	185	149	81.9	74.1
		Moderate	5	496	148	103	65.5	51.2
		High	7	350	109	74	48.2	36.8

The proportion of cases in 2017 and 2018 identified in the clusters of 2014–2016 by heatmap radius and cluster density

^1^Cluster density based on the cut-off values ≥ 2 (low density), ≥ 5 (moderate density), and ≥ 10 (high density)

## Discussion

This study identified significant clustering of cases in Pamekasan and Pasuruan using the heatmap methodology with 15 combinations of radii and cluster density. The proportion of total cases in clusters increased with heatmap radius and ranged from 3 to almost 100%. The proportion of population (in clusters) targeted for PEP decreased with heatmap radius from > 100 to 3%. Moreover, our analysis showed that the heatmap tool is practical. A substantial percentage of the leprosy cases in 2017 and 2018 was localized in the identified clusters established in previous years (21% when using the 500 m and low-density cluster setting, and almost 90% of the leprosy cases of 2017 and 2018 using the 2000 m cluster setting). This would imply that between 21 and 90% of the leprosy cases living in targeted clusters might be prevented in the next years.

We found a considerable higher NCDR in the northern subdistricts in Pamekasan and Pasuruan indicating that more transmission of *M. leprae* is taking place in these subdistricts. Individuals would have a higher risk to develop leprosy in these subdistricts and therefore may need to be targeted with PEP interventions first. If the new cases would be evenly distributed throughout the whole area, i.e., if there were small or no differences in NCDR between subunits, the entire area should be considered as a target area.

Our findings showed a more clustered distribution of leprosy cases in Pasuruan than in Pamekasan (Morans’ I value 0.44 and 0.27 respectively). Also, the proportion of total cases in clusters in Pasuruan is higher overall than in Pamekasan for clusters based on high density, except for the 500 m radius. The strength of clustering may determine whether a higher or lower radius should be selected to determine clusters that would be targeted for prophylactic interventions. When the distribution of cases is more diffuse, it would be more effective to define clusters using cluster settings with larger radii and low density clustering. In areas with a focused distribution of cases, cluster settings with low radii and moderate to high-density clustering are recommended. In general, smaller clusters or a small total cluster area may be preferred, as they are operationally easier to manage. However, choosing for smaller clusters also means that the proportion of contacts targeted with PEP among those who may benefit from PEP will also be smaller.

The importance of more accurate information on spatial heterogeneous distribution of disease and endemicity level at sub-district or village level is recognized by program managers of neglected tropical diseases as an added value to determine the target populations for preventive interventions [36, 37]. For example, schistosomiasis and soil-transmitted helminths show a heterogeneous distribution with large clusters of cases located near infested water and soil, respectively. Since the whole population in these areas is considered to be at risk, a population-wide strategy (i.e., mass drug administration) is recommended in that area [37]. Leprosy also shows a heterogeneous distribution but with small clusters of cases. Therefore, a cluster-based PEP strategy targeting only those individuals that might have been in contact with a leprosy patient is considered more effective than targeting the whole population in the district.

In this study, we considered two contact-based PEP strategies, (I) providing PEP to 20 contacts per leprosy case, and (II) providing PEP to 100 individuals per leprosy case, and one population-wide PEP strategy, (III) providing PEP to the total population in clusters. Contact-based strategies include tracing and screening contacts of a leprosy case for leprosy signs and symptoms. Those who have no leprosy or other contraindications receive PEP. Strategy I, wherein 20 close contacts (e.g., household, neighbours, and social contacts) are screened has shown to be effective and feasible in a large study [5]. In very high endemic areas, however, this strategy may not be sufficient to significantly reduce the transmission because a much larger proportion of the population could be considered a contact. Increasing the number of contacts or a population-wide strategy may be more effective. Strategy II has not been studied yet, but a modeling study showed that a larger reduction in incidence could be achieved if more contacts were included for screening and PEP [6]. Strategy III would resemble a blanket or population strategy in high risk areas. A study by Bakker (2005) showed a significant decline in incidence in the first three years after providing PEP to all eligible persons on three islands using a blanket approach compared to the island where only close contacts received PEP and the control island [38]. The choice of PEP strategy also depends on available resources. We calculated that the total number of individuals requiring PEP in the two districts ranged from 580 to 22,740 using strategy I, from 2900 to 113,700 using strategy II, and from 256 to 877,413 using strategy III. With limited resources (i.e., small budget and limited trained health staff) strategy I is more suitable, while strategy II or III should only be considered if sufficient resources are available. All three strategies target the cases in clusters, but the contacts of cases outside a cluster should also be targeted with PEP as recommended by the WHO guidelines (at least the household contacts) [4].

We observed that the proportion of population targeted for PEP exceeded 100% in five clusters settings with a small radius (i.e., ≤ 1000 m) for strategy I and II because the identified cluster area is relatively small and covers small populations. Therefore, the calculated number of individuals targeted for PEP may exceed the total population size in these small clusters. This implies that certain cluster settings are not suitable to facilitate PEP strategies that have a fixed number of contacts or individuals per index (i.e., strategy I or II). In this case, the program manager could also consider targeting the total population in the cluster only (i.e., strategy III). Another option is to use a cluster setting with a larger radius (> 1000 m) and lower density, which would result in a larger cluster area and population.

Based on this study, program managers may use the heatmap methodology to develop a practical guideline to determine target populations with PEP. An example of how such a guideline could be set up can be found in Additional file 1: File. S3. To develop such a guideline, the following three components should be available. Firstly, a dataset of the GPS locations of leprosy patients at household level (i.e., exact location of resident or within 10–20 m of the resident) to identify more accurate clusters. GPS coordinates at village level (i.e., centre of village with total number of cases in the village) can also be used but will result in more aggregated clusters. GPS locations at a higher level are not preferred because it will result in large clusters. Secondly, the location and population data of villages to calculate the population density per village and proportion of population targeted for PEP. In this study, the proportion of population is based on the density per km^2^ as calculated for the whole district because the population data at a subunit level was not available or complete. In the most urban or remote areas the density per km square will differ considerably and therefore, the proportions to be targeted in these clusters are less accurate and not comparable. When population data per village are available the proportions will be more accurate. Thirdly, an estimation should be at hand of the resources available in the area, either in terms of funding and/or in terms of trained health staff.

The hotspot analysis used in this study has proven to be a practical method to identify clusters of cases of disease and determine the target populations for interventions. Our sensitivity analysis showed that between 21 and 90% of the cases of the next years fall in a defined cluster. This may indicate that in high-endemic areas with a stable leprosy incidence, clusters will remain in the same areas over the next years [22]. Interventions should therefore be prioritized in these clusters.

A possible limitation of our approach is that the cut-off values to define low, moderate, and high-density clustering were chosen arbitrarily. Alternatively, in a study to identify hotspots of malaria cases for each season in Myanmar, the median density was used given a fixed radius to identify clusters [39]. We did not use the median density value because we varied the radius (500 to 2500 m), which would have resulted in a different median density value for each radius. Since our aim was to directly compare the implication of choices regarding radii and density, we selected three cut-off values of density that would be simple and meaningful for a policymaker. Also, our approach has only been used in the context of two high-endemic districts in Indonesia and may not completely apply to other (foreign) areas. For example, depending on the size of districts, one may need to increase or decrease radii to identify clusters of reasonable size. Nevertheless, the general concept of this approach may apply to all contexts.

Another consideration is that we did not take into account urban and rural areas and assumed that the population is evenly distributed in a district, whereas, in reality, this is not the case. This also could partly explain why in small clusters the proportion targeted with PEP exceeds 100%. If the actual population per village or city is known a more accurate estimate of the target population can be made. Also, the distance between leprosy cases may be different in urban and rural areas. Therefore, program managers may need to consider different cluster settings, for example, a small radius in urban areas and a large radius in very remote areas.

## Conclusion

The choice of parameters used in the hotspot analysis has a direct effect on the proportion of cases that is considered part of any cluster and thus the total number of contacts or individuals that would be included in case detection and prophylactic interventions. Knowledge on the strength of clustering, population at village level as well as operational factors can guide policy and management choices related to leprosy control programs to identify high-risk clusters and estimate the number of people targeted for prophylactic interventions.

## Supplementary Information

**Additional file 1: Fig. S1**. Heatmap of leprosy cases in Pamekasan and Pasuruan. Heatmap of leprosy cases using a 1500 m radius and singleband pseudocolor yellow to brown for Pamekasan (left) and Pasuruan (right). A darker colour indicates higher density and value. **Fig. S2.** Cluster maps of leprosy cases registered from 2014 to 2016 of Pamekasan and Pasuruan. Cluster maps of leprosy cases registered from 2014 to 2016 of Pamekasan (left) and Pasuruan (right) by heatmap radius and cluster density. Heatmap radius varies from 500 m (top row) to 2500 m (bottom row). The blue colour represents low density clusters, the orange colour represents moderate density clusters, and the green colour represents high density clusters. **File S3. **An example of how a guideline can be set up to select the heatmap radius and cluster density. An example of a diagram to select the heatmap radius and cluster density (green) in three steps: distribution of cases in the area based on Moran’s I value (orange), the preferred proportion of total leprosy cases in clusters (yellow) and the PEP strategy either 20 contacts, 100 individuals or population-wide (green: left, middle and right column respectively). The presented cluster setting recommendations are selected using three criteria: i) proportion of cases in clusters, ii) total population to target, and iii) size of cluster area. We selected the cluster settings with the highest as possible proportion of cases within that specific range first (Table 1 of main manuscript). Then, in case of 2 of more settings with similar proportions, we selected the setting with the largest proportion of population living in clusters that would be targeted for PEP and smallest cluster area.

## Data Availability

The datasets generated and/or analysed during the current study are not publicly available due to identifiable data of persons affected by leprosy but are available from the corresponding author on motivated request.

## Abbreviations

GIS: Geographic information systems
GPS: Global positioning system
LPEP: Leprosy post-exposure prophylaxis
NCD: New case detection
NCDR: New case detection rate
PEP: Post-exposure prophylaxis
SDR-PEP: Single dose rifampicin post-exposure prophylaxis
