# Supplementary material for: Determining target populations for leprosy prophylactic interventions: a hotspot analysis in Indonesia
Source: BMC Infect Dis. 2022 Feb 7;22:131. doi: 10.1186/s12879-022-07103-0 (PMC8822733; doi:10.1186/s12879-022-07103-0)
Supplement: Supplementary file 1 — Additional file 1: Fig. S1. Heatmap of leprosy cases in Pamekasan and Pasuruan. Heatmap of leprosy cases using a 1500 m radius and singleband pseudocolor yellow to brown for Pamekasan (left) and Pasuruan (right). A darker colour indicates higher density and value. Fig. S2. Cluster maps of leprosy cases registered from 2014 to 2016 of Pamekasan and Pasuruan. Cluster maps of leprosy cases registered from 2014 to 2016 of Pamekasan (left) and Pasuruan (right) by heatmap radius and cluster density. Heatmap radius varies from 500 m (top row) to 2500 m (bottom row). The blue colour represents low density clusters, the orange colour represents moderate density clusters, and the green colour represents high density clusters. File S3. An example of how a guideline can be set up to select the heatmap radius and cluster density. An example of a diagram to select the heatmap radius and cluster density (green) in three steps: distribution of cases in the area based on Moran’s I value (orange), the preferred proportion of total leprosy cases in clusters (yellow) and the PEP strategy either 20 contacts, 100 individuals or population-wide (green: left, middle and right column respectively). The presented cluster setting recommendations are selected using three criteria: i) proportion of cases in clusters, ii) total population to target, and iii) size of cluster area. We selected the cluster settings with the highest as possible proportion of cases within that specific range first (Table 1 of main manuscript). Then, in case of 2 of more settings with similar proportions, we selected the setting with the largest proportion of population living in clusters that would be targeted for PEP and smallest cluster area. [file 12879_2022_7103_MOESM1_ESM.docx]

Supplementary files

Fig. S1 Heatmap of leprosy cases in Pamekasan and Pasuruan.

Fig. S2 Cluster maps of leprosy cases registered from 2014 to 2016 of Pamekasan and Pasuruan.

File S3 How a guideline can be set up to select the heatmap radius and cluster density.


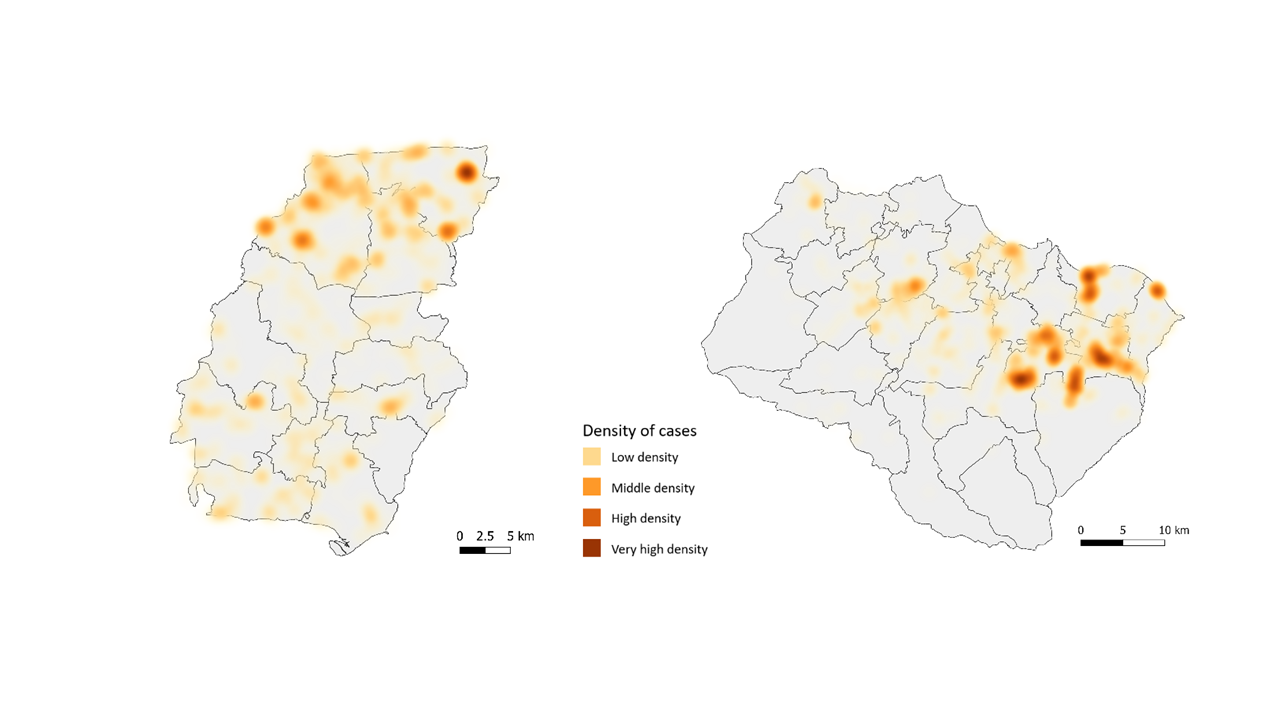


**Fig. S1 Heatmap of leprosy cases using a 1500 m radius and singleband pseudocolor yellow to brown for Pamekasan (left) and Pasuruan (right). A darker colour indicates higher density and value.**

**
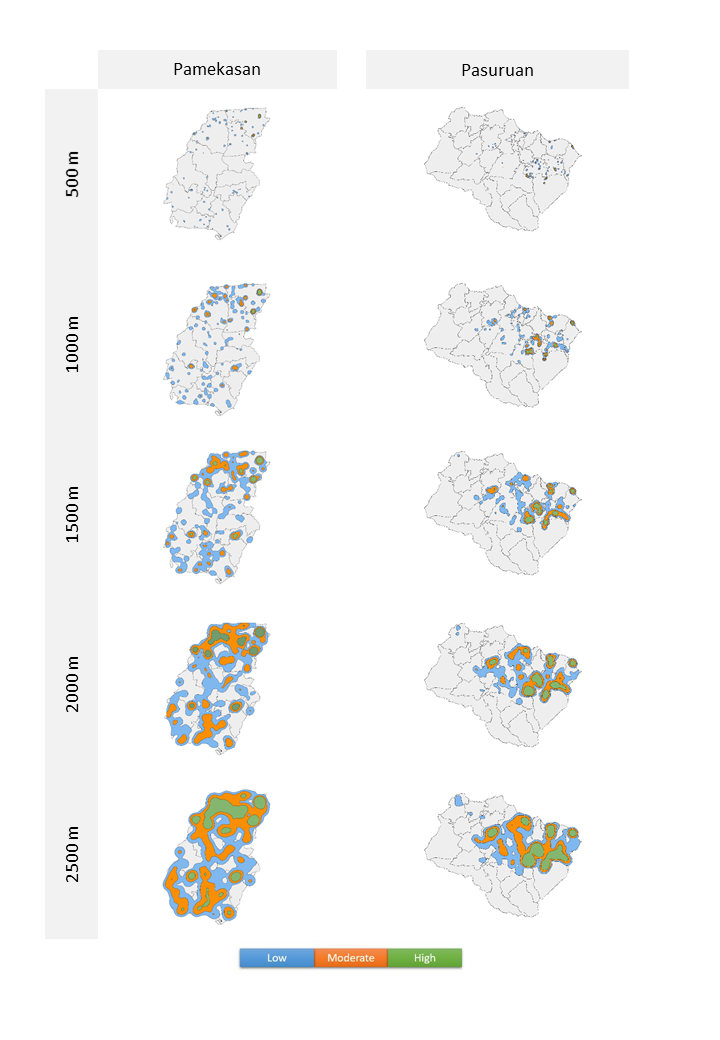
**

**Fig S2 Cluster maps of leprosy cases registered from 2014 to 2016 of Pamekasan (left) and Pasuruan (right) by heatmap radius and cluster density. Heatmap radius varies from 500 meter (top row) to 2500 meter (bottom row). The blue colour represents low density clusters, the orange colour represents moderate density clusters, and the green colour represents high density clusters.**

**File S3 How a guideline can be set up to select the heatmap radius and cluster density.**

**Steps**

1. Define the project area (e.g., country, state, province, district), the period (years) of leprosy cases to be collected and analyzed, and the level of data (e.g., household, village).
2. Upload all data points, project area boundaries and population data in QGIS.
3. Calculate the NCDR per subunit (e.g., village, neighbourhood, subdistrict) per 1,000,000 population.
4. Calculate the Moran’s I value in ClusterSeer using the tool ‘Univariate Moran’s I’.
5. Start at the left side of the diagram. Define the distribution of cases using the Moran’s I value. With a positive value of 0.4 and above select ‘focused’, with a value below 0.4 select ‘diffused’.
6. Select the proportion of total leprosy cases in clusters whose contacts you wish to target for PEP: ‘< 25%’, ‘25-50%’, ‘50-75%’, or ‘> 75%’.
7. Use the cluster settings recommended in the hotspot analysis to identify the clusters targeted for each PEP strategy.
8. Calculate the number of individuals targeted for PEP for each strategy:
9. 20 contacts: multiply total number of cases in clusters by 20
10. 100 individuals: multiply total number of cases in clusters by 100
11. population-wide: multiply the cluster area (km^2^) by population density
12. Select the final cluster setting and PEP strategy based on available resources.

**An example of a diagram to select the heatmap radius and cluster density (green) in three steps: distribution of cases in the area based on Moran’s I value (orange), the preferred proportion of total leprosy cases in clusters (yellow) and the PEP strategy either 20 contacts, 100 individuals or population-wide (green: left, middle and right column respectively). The presented cluster setting recommendations are selected using three criteria: i) proportion of cases in clusters, ii) total population to target, and iii) size of cluster area. We selected the cluster settings with the highest as possible proportion of cases within that specific range first (Table 1 of main manuscript). Then, in case of 2 of more settings with similar proportions, we selected the setting with the largest proportion of population living in clusters that would be targeted for PEP and smallest cluster area.**


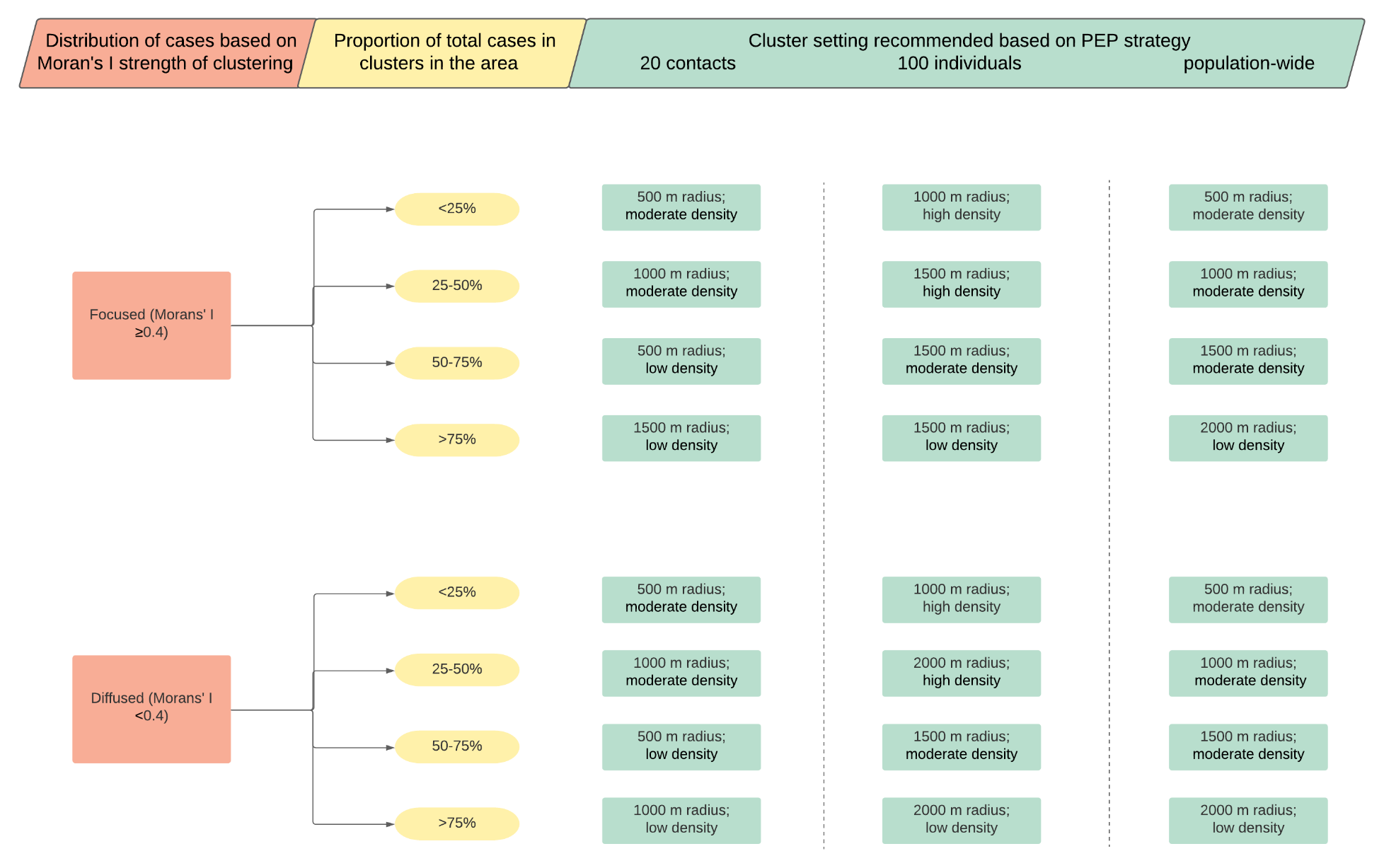


Practical example of using the proposed guideline

A leprosy programme manager (LPM) would like to use the guideline to implement targeted PEP interventions in all the subdistricts in Pamekasan because all sub-districts are endemic for leprosy.

Local staff have collected the GPS coordinates of all leprosy patients and the programme manager or GIS specialist performed the Moran’s I in ClusterSeer resulting in a value of 0.3. Based on this result the LPM should select the ‘diffused’ arm in the orange column.

The LPM decides that a maximum of 70% of the leprosy cases should be in a cluster and thus targeted for extensive preventive intervention. Based on this decision, the 50-75% arm is selected in the yellow column.

The LPM or GIS specialist will identify the clusters based on the cluster setting recommendation of each strategy (20 contacts: 500 m and low density; 100 individuals and population-wide: 1500 m and moderate density), and then calculate the number of people targeted with PEP for all three strategies. This would result in 12,880 individuals, if 20 contacts are targeted, 75,800 individuals, if 100 individuals are targeted, and 187,031 individuals, if whole cluster (population-wide) is targeted.

The LMP indicates that resources only allow targeting around 15,000 people. Based on the calculations, the strategy targeting 20 contacts is recommended using a cluster setting 500 m and low density.
